# Use of 15-Valent Pneumococcal Conjugate Vaccine and 20-Valent Pneumococcal Conjugate Vaccine Among U.S. Adults: Updated Recommendations of the Advisory Committee on Immunization Practices — United States, 2022

**DOI:** 10.15585/mmwr.mm7104a1

**Published:** 2022-01-28

**Authors:** Miwako Kobayashi, Jennifer L. Farrar, Ryan Gierke, Amadea Britton, Lana Childs, Andrew J. Leidner, Doug Campos-Outcalt, Rebecca L. Morgan, Sarah S. Long, H. Keipp Talbot, Katherine A. Poehling, Tamara Pilishvili

**Affiliations:** ^1^National Center for Immunization and Respiratory Diseases, CDC; ^2^Epidemic Intelligence Service, CDC; ^3^CDC Foundation; ^4^University of Arizona, College of Medicine, Phoenix, Arizona; ^5^Department of Health Research Methods, Evidence and Impact, McMaster University, Hamilton, Ontario; ^6^Drexel University College of Medicine, Philadelphia, Pennsylvania; ^7^Vanderbilt University School of Medicine, Nashville, Tennessee; ^8^Wake Forest School of Medicine, Winston-Salem, North Carolina.

In 2021, 20-valent pneumococcal conjugate vaccine (PCV) (PCV20) (Wyeth Pharmaceuticals LLC, a subsidiary of Pfizer Inc.) and 15-valent PCV (PCV15) (Merck Sharp & Dohme Corp.) were licensed by the Food and Drug Administration for adults aged ≥18 years, based on studies that compared antibody responses to PCV20 and PCV15 with those to 13-valent PCV (PCV13) (Wyeth Pharmaceuticals LLC, a subsidiary of Pfizer Inc.). Antibody responses to two additional serotypes included in PCV15 were compared to corresponding responses after PCV13 vaccination, and antibody responses to seven additional serotypes included in PCV20 were compared with those to the 23-valent pneumococcal polysaccharide vaccine (PPSV23) (Merck Sharp & Dohme Corp.). On October 20, 2021, the Advisory Committee on Immunization Practices (ACIP) recommended use of either PCV20 alone or PCV15 in series with PPSV23 for all adults aged ≥65 years, and for adults aged 19–64 years with certain underlying medical conditions or other risk factors* who have not previously received a PCV or whose previous vaccination history is unknown. ACIP employed the Evidence to Recommendation (EtR) framework,^†^ using the Grading of Recommendations, Assessment, Development and Evaluation (GRADE)^§^ approach to guide its deliberations regarding use of these vaccines. Before this, PCV13 and PPSV23 were recommended for use for U.S. adults and the recommendations varied by age and risk groups. This was simplified in the new recommendations.

PPSV23 has been recommended for use in the United States since the 1980s for adults aged ≥65 years and for younger adults with underlying conditions that increase their risk for pneumococcal disease (*1*). PCV13 was first recommended for use in U.S. children in 2010, and indirect effects from its use in children reduced PCV13-type pneumococcal disease incidence in all adult groups (Figure). In 2012, ACIP recommended administration of PCV13 in series with PPSV23 for adults with immunocompromising conditions,^¶^ cerebrospinal fluid leaks, or cochlear implants (*2*), and in 2014, the recommendation was extended to all adults aged ≥65 years (*3*). On the basis of review of accrued evidence, the PCV13 recommendation was changed in 2019 to shared clinical decision-making for adults aged ≥65 years without an immunocompromising condition, cerebrospinal fluid leak, or cochlear implant. The recommended pneumococcal vaccine doses and intervals between doses differ by age and underlying conditions, making adult pneumococcal vaccine recommendations complicated.

**FIGURE:**
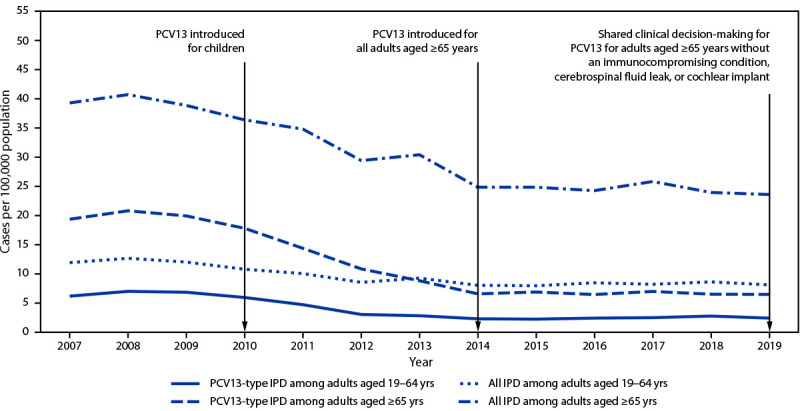
Incidence of all invasive pneumococcal disease and 13-valent pneumococcal conjugate vaccine-type* invasive pneumococcal disease among adults aged ≥19 years, by invasive pneumococcal disease type and age group — United States, 2007–2019^†^ **Abbreviations:** IPD = invasive pneumococcal disease; PCV13 = 13-valent pneumococcal conjugate vaccine. * Includes serotype 6C, which shows cross-protection from 6A antigen in PCV13 and was grouped with PCV13 serotypes for IPD incidence. ^†^ Active Bacterial Core surveillance, 2021.

Recent systematic reviews continue to support the effectiveness of PCV13 against invasive pneumococcal disease (IPD)** and pneumococcal pneumonia among adults (*4*,*5*). Whereas effectiveness of PPSV23 against IPD has been demonstrated, data on effectiveness against pneumococcal pneumonia were considered to be inconsistent (*3*); recent observational studies reported 21%–46% effectiveness against PPSV23-type pneumococcal pneumonia when PPSV23 was given <5 years before illness onset (*6*–*8*). Nevertheless, older adults and adults with chronic medical conditions^††^ or immunocompromising conditions, cerebrospinal fluid leaks, or cochlear implants (certain underlying conditions) remain at increased risk for pneumococcal disease, accounting for >90% of adult IPD cases in 2019 (Active Bacterial Core surveillance, unpublished data, 2021).

During February–October 2021, ACIP reviewed the epidemiology of pneumococcal disease and considerations for use of PCV15 and PCV20 in adults. The ACIP Pneumococcal Vaccines Work Group (Work Group) evaluated the quality of evidence for PCV15 and PCV20 immunogenicity and safety using the GRADE approach.^§§^ Using the EtR framework,^¶¶^ the Work Group reviewed relevant scientific evidence regarding the benefits and harms of PCV15 and PCV20 use among adults aged ≥65 years and younger adults with certain underlying conditions. Within the EtR framework, ACIP considered the importance of the public health problem, benefits and harms, target populations’ values and preferences, resource use, equity, acceptability, and feasibility for PCV15 or PCV20 use. After a systematic review of the literature, the Work Group defined critical outcomes and used GRADE to assess certainty of evidence rated on a scale of 1 (high certainty) to 4 (very low certainty) (*9*).

## Evidence

**Pneumococcal disease incidence in adults.** During 2018–2019, the incidence of all IPD in adults aged ≥65 years was 24 per 100,000 population (Figure), and PCV13 serotypes accounted for 27% of cases; additional serotypes unique to PCV15,*** PCV20,^†††^ and PPSV23^§§§^caused 15%, 27%, and 35% of IPD, respectively. In adults aged 19–64 years with certain underlying conditions, PCV13 serotypes accounted for 30% of IPD; serotypes unique to PCV15, PCV20, and PPSV23 caused 13%, 28%, and 43% of IPD, respectively. Estimates of pneumococcal pneumonia incidence are more variable. Annual incidence among U.S. adults aged <65 and ≥65 years hospitalized with community-acquired pneumonia was estimated at 126–422 and 847–3,365 per 100,000, respectively, during 2010–2016 (*10*). In a multisite study of adults hospitalized with community-acquired pneumonia, 4.6% of cases were caused by PCV13 serotypes, and 1.4% and 3.3% were caused by additional serotypes included in PCV15 and PCV20, respectively (*11*).

**PCV15 immunogenicity.** PCV15 contains pneumococcal polysaccharide serotypes 22F and 33F in addition to the PCV13 serotypes, conjugated to CRM197 (genetically detoxified diphtheria toxin) (*9*). Phase II and III randomized controlled trials (RCTs) evaluated the immunogenicity and safety of a dose of PCV15 compared with a dose of PCV13 in healthy adults aged ≥50 years (*12*–*14*), adults aged 18–49 years who are Native American (a population with higher rates of IPD than the general U.S. population) (*15*) or with ≥1 risk condition for pneumococcal disease (*16*), and adults aged ≥18 years with HIV infection (*17*). Serotype-specific functional antibody responses were measured 1 month after vaccination using an opsonophagocytic activity (OPA) assay. Correlates of protection have not been established for adults. In one phase III RCT among adults aged ≥50 years, PCV15 met the noninferiority criteria^¶¶¶^ compared with PCV13 for the 13 shared serotypes and had statistically significantly greater response**** for shared serotype 3 and PCV15-unique serotypes 22F and 33F (*14*). In studies that evaluated the immunogenicity of PCV15 or PCV13 followed by PPSV23 2–12 months later (*16*–*18*), persons who received PCV15 had numerically similar or higher OPA geometric mean antibody titers (GMTs) for 9–13^††††^ shared PCV13 serotypes and a higher percentage of seroresponders^§§§§^ for 5–11 shared serotypes compared with persons who received PCV13 when measured 1 month after receipt of PPSV23.

**PCV15 safety.** Safety of PCV15 was assessed in seven RCTs with 5,630 participants aged ≥18 years who received 1 dose of PCV15. Most participants were immunocompetent; however, one study included 302 adults with HIV infection. Participants included those vaccinated with PPSV23 ≥1 year before receiving PCV15, those who received PCV15 followed by PPSV23, and those who received PCV15 concomitantly with a seasonal inactivated quadrivalent influenza vaccine (QIV). The most frequently reported adverse reactions were injection site pain, fatigue, and myalgia. The rates of serious adverse events (SAEs) within 6 months of vaccination were 2.5% among PCV15 recipients and 2.4% among PCV13 recipients. No SAEs or deaths were considered to be related to the study vaccines (*9*,*19*).

**PCV20 immunogenicity.** PCV20 contains pneumococcal polysaccharide serotypes 8, 10A, 11A, 12F, 15B, 22F, and 33F, in addition to PCV13 serotypes, conjugated to CRM197 (*20*). A phase II study among adults aged 60–64 years and two phase III RCTs among adults aged ≥18 years evaluated immunogenicity and safety of PCV20 compared with PCV13 and with PPSV23 for the seven additional serotypes included in PCV20 (*21*–*23*). These studies included adults with stable medical conditions, but none included adults with immunocompromising conditions. Compared with PCV13 recipients, PCV20 recipients elicited responses that met noninferiority criteria^¶¶¶¶^ for all 13 serotypes in a phase III trial among adults aged ≥60 years (*21*); however, PCV20 recipients appeared to have lower GMTs and included a lower percentage of seroresponders to 12–13 of the 13 PCV13-shared serotypes (*21*,*22*). Compared with PPSV23 recipients, PCV20 recipients had numerically higher GMTs and a higher percentage of seroresponders to six of seven (excluding serotype 8) shared non-PCV13 serotypes (*21*,*23*); noninferiority criteria were met for those six serotypes (*21*).

**PCV20 safety.** Safety of PCV20 was assessed in six trials among immunocompetent adults aged ≥18 years that included a total of 4,552 participants who received PCV20. Participants included those who were naïve to pneumococcal vaccination and those who had previously received pneumococcal vaccination. The most frequently reported adverse reactions were injection site pain, muscle pain, fatigue, headache, and joint pain. SAEs reported within 6 months after vaccination occurred among 1.5% of PCV20 recipients and 1.8% among controls. No SAEs or deaths were considered to be related to study vaccines (*20*,*24*).

**Intervals between PCV and PPSV23.** Findings from eight immunogenicity studies that evaluated the immune response after a sequence of 7-valent PCV, PCV13, or PCV15 followed by PPSV23 administered at intervals of 2, 6, or 12 months or 3–4 years were reviewed (*16*–*18*,*25*–*29*). Three studies comparing intervals ranging from 2 to 6 months between administration of PCV and PPSV23 found no significant difference in immunogenicity measured after PPSV23 receipt, although reactogenicity tended to be higher with shorter intervals (*25*–*29*). In a study that compared antibody responses to 1 dose of PCV13 with responses to PCV13 followed by PPSV23 1 year apart, the immune responses following PPSV23 were significantly lower compared with the responses after a dose of PCV13 for eight of 12 common serotypes (*27*). In another study that compared antibody response to 1 dose of PCV13 with responses to PCV13 followed by PPSV23 approximately 4 years apart, the immune responses following PPSV23 were significantly higher for seven of 12 common serotypes (*26*). These findings suggested that longer intervals between administration of PCV and PPSV23 might improve immunogenicity in immunocompetent adults, although a direct comparison between a 1- versus 4-year interval was not made.

**Cost-effectiveness.** Economic models assessed cost-effectiveness of the new policy options compared with existing recommendations (*30*). Three economic models assessed PCV20 alone for all adults aged ≥65 years; cost-effectiveness estimates ranged from cost-saving^*****^ to $39,000 per quality-adjusted life-year (QALY) gained. Two economic models assessed use of PCV15 in series with PPSV23 for all adults aged ≥65 years; estimates ranged from cost-saving to $282,000 per QALY gained. The CDC model found cost savings in all scenarios for use of either PCV20 alone or PCV15 in series with PPSV23 for all adults aged ≥65 years. Cost estimates of policy options for adults aged 19–64 years with certain underlying medical conditions ranged from $11,000 to $292,000 per QALY gained for PCV20 and from $250,000 to $656,000 for PCV15 in series with PPSV23.

**Summary.** Use of PCV20 alone or PCV15 in series with PPSV23 is expected to reduce pneumococcal disease incidence in adults aged ≥65 years and in those aged 19–64 years with certain underlying conditions. Findings from studies suggested that the immunogenicity and safety of PCV20 alone or PCV15 in series with PPSV23 were comparable to PCV13 alone or PCV13 in series with PPSV23. Cost-effectiveness studies demonstrated that use of PCV20 alone or PCV15 in series with PPSV23 for adults at age 65 years was cost-saving. The new policy simplifies adult pneumococcal vaccine recommendations (Table 1) and is expected to improve vaccine coverage among adults and prevent more pneumococcal disease. An amendment to recommend PCV20 for all adults aged ≥50 years instead of age ≥65 years was considered but rejected (Table 2). A summary of Work Group deliberations on use of either PCV20 alone or PCV15 in series with PPSV23 for all adults aged ≥65 years or adults aged 19–64 years with certain underlying conditions is available in the EtR tables.

**TABLE 1:** Recommendations for use of 15-valent pneumococcal conjugate vaccine in series with 23-valent pneumococcal polysaccharide vaccine or 20-valent pneumococcal conjugate vaccine in pneumococcal conjugate vaccine-naïve adults aged ≥19 years — United States, 2022

Medical indication group	Specific underlying medical condition	Age group, yrs	
		19–64	≥65
None	None	None	1 dose of PCV20 or 1 dose of PCV15 followed by a dose of PPSV23 ≥1 years later*
Underlying medical conditions or other risk factors	Alcoholism	1 dose of PCV20 or 1 dose of PCV15 followed by a dose of PPSV23 ≥1 years later^§^	1 dose of PCV20 or 1 dose of PCV15 followed by a dose of PPSV23 ≥1 years later*
	Chronic heart disease^†^		
	Chronic liver disease		
	Chronic lung disease^¶^		
	Cigarette smoking		
	Diabetes mellitus		
	Cochlear implant		
	CSF leak		
	Congenital or acquired asplenia		
	Sickle cell disease or other hemoglobinopathies		
	Chronic renal failure**		
	Congenital or acquired immunodeficiencies**^,††^		
	Generalized malignancy**		
	HIV infection**		
	Hodgkin disease**		
	Iatrogenic immunosuppression**^,§§^		
	Leukemia**		
	Lymphoma**		
	Multiple myeloma**		
	Nephrotic syndrome**		
	Solid organ transplant**		

**Abbreviations**: CSF = cerebrospinal fluid; PCV15 =15-valent pneumococcal conjugate vaccine; PCV20 = 20-valent pneumococcal conjugate vaccine; PPSV23 = 23-valent pneumococcal polysaccharide vaccine.

* Adults with immunocompromising conditions, cochlear implant, or CSF leak might benefit from shorter intervals such as ≥8 weeks. These vaccine doses do not need to be repeated if given before age 65 years.

^†^ Includes congestive heart failure and cardiomyopathies.

^§^ Adults with immunocompromising conditions, cochlear implant, or CSF leak might benefit from shorter intervals such as ≥8 weeks.

^¶^ Includes chronic obstructive pulmonary disease, emphysema, and asthma.

** Indicates immunocompromising conditions.

^††^ Includes B- (humoral) or T-lymphocyte deficiency, complement deficiencies (particularly C1, C2, C3, and C4 deficiencies), and phagocytic disorders (excluding chronic granulomatous disease).

^§§^ Diseases requiring treatment with immunosuppressive drugs, including long-term systemic corticosteroids and radiation therapy.

**TABLE 2:** Age-based policy options for use of 15-valent pneumococcal conjugate vaccine or 20-valent pneumococcal conjugate vaccine in adults presented for a vote and considerations by the Advisory Committee on Immunization Practices — United States, October 2021

Proposed policy	Considerations raised during October 2021 ACIP meeting in favor of the option	Outcome (votes in favor: against)
Adults aged ≥50 years who have not previously received a pneumococcal conjugate vaccine or whose previous vaccination history is unknown should receive a pneumococcal conjugate vaccine (either PCV20 or PCV15). If PCV15 is used, this should be followed by a dose of PPSV23.	Might reduce existing pneumococcal disease disparity in adults aged 50–64 years.	Rejected (4:11)
	Age-based recommendation is easier to implement than risk-based recommendation.	
	Might provide more opportunities to vaccinate adults before underlying conditions develop.	
Adults aged ≥65 years who have not previously received a pneumococcal conjugate vaccine or whose previous vaccination history is unknown should receive a pneumococcal conjugate vaccine (either PCV20 or PCV15). If PCV15 is used, this should be followed by a dose of PPSV23.	Potential for waning vaccine-induced immunity makes it favorable to vaccinate later in life when risk for disease is higher.	Affirmed (15:0)
	Consistently cost saving in cost-effectiveness analyses.	
	Still provides an opportunity for higher PCV coverage in adults compared with current recommendations.	
	No evidence that lowering the age-based recommendation will reduce disparity in vaccine-preventable disease compared with risk-based recommendations.	

**Abbreviations:** ACIP = Advisory Committee on Immunization Practices; PCV = pneumococcal conjugate vaccine ; PCV15 = 15-valent PCV; PCV20 = 20-valent PCV; PPSV23 = 23-valent pneumococcal polysaccharide vaccine.

## New Pneumococcal Vaccine Recommendations

**Adults aged ≥65 years.** Adults aged ≥65 years who have not previously received PCV or whose previous vaccination history is unknown should receive 1 dose of PCV (either PCV20 or PCV15). When PCV15 is used, it should be followed by a dose of PPSV23 (Table 1).

**Adults aged 19–64 years with certain underlying medical conditions or other risk factors.** Adults aged 19–64 years with certain underlying medical conditions or other risk factors who have not previously received PCV or whose previous vaccination history is unknown should receive 1 dose of PCV (either PCV20 or PCV15). When PCV15 is used, it should be followed by a dose of PPSV23.

## Clinical Guidance

**Dosing schedule.** When PCV15 is used, the recommended interval between administration of PCV15 and PPSV23 is ≥1 year. A minimum interval of 8 weeks can be considered for adults with an immunocompromising condition, cochlear implant, or cerebrospinal fluid leak to minimize the risk for IPD caused by serotypes unique to PPSV23 in these vulnerable groups (*31*).

**Adults with previous PPSV23 only.** Adults who have only received PPSV23 may receive a PCV (either PCV20 or PCV15) ≥1 year after their last PPSV23 dose. When PCV15 is used in those with history of PPSV23 receipt, it need not be followed by another dose of PPSV23.

**Adults with previous PCV13.** The incremental public health benefits of providing PCV15 or PCV20 to adults who have received PCV13 only or both PCV13 and PPSV23 have not been evaluated. These adults should complete the previously recommended PPSV23^†††††^ series (*2*,*30*).

**Coadministration with other vaccines.** PCV15, PCV20, or PPSV23 can be coadministered with QIV in an adult immunization program, as concomitant administration (PCV15 or PPSV23 and QIV [Fluarix], PCV20 and adjuvanted QIV [Fluad]) has been demonstrated to be immunogenic and safe. However, slightly lower pneumococcal serotype-specific OPA GMTs or geometric mean concentrations were reported when pneumococcal vaccines were coadministered with QIV compared with when pneumococcal vaccines were given alone (*9*,*19*,*32*,*33*). Currently, no data are available on coadministration with other vaccines (e.g., tetanus, diphtheria, acellular pertussis vaccine, hepatitis B, or zoster vaccine) among adults. Evaluation of coadministration of PCV15, PCV20, or PPSV23 with COVID-19 vaccines is ongoing (*34*,*35*).

## Future Research and Monitoring Priorities

CDC and ACIP will continue to assess safety of PCV15 and PCV20 vaccines, monitor the impact of implementation of new recommendations, and assess postimplementation vaccine effectiveness and update pneumococcal vaccination recommendations as appropriate.

Before administering PCV20, PCV15, or PPSV23, health care providers should consult relevant package inserts (*9*,*20*,*36*) regarding precautions and contraindications. Adverse events occurring after administration of any vaccine should be reported to the Vaccine Adverse Event Reporting System (VAERS). Reports can be submitted to VAERS online, by fax, or by mail. Additional information about VAERS is available at https://vaers.hhs.gov/.

**Summary**

**What is already known about this topic?**

Currently, the 13-valent pneumococcal conjugate vaccine (PCV) (PCV13) and the 23-valent pneumococcal polysaccharide vaccine (PPSV23) are recommended for U.S. adults. Recommendations vary by age and risk groups.

**What is added by this report?**

On October 20, 2021, the Advisory Committee on Immunization Practices recommended 15-valent PCV (PCV15) or 20-valent PCV (PCV20) for PCV–naïve adults who are either aged ≥65 years or aged 19–64 years with certain underlying conditions. When PCV15 is used, it should be followed by a dose of PPSV23, typically ≥1 year later.

**What are the implications for public health practice?**

Pneumococcal vaccination recommendations were simplified across age and risk group. Eligible adults may receive either PCV15 in series with PPSV23 or PCV20 alone.

## Footnotes

* Alcoholism; chronic heart, liver, or lung disease; chronic renal failure; cigarette smoking; cochlear implant; congenital or acquired asplenia; cerebrospinal fluid leak; diabetes mellitus; generalized malignancy; HIV; Hodgkin disease; immunodeficiency; iatrogenic immunosuppression; leukemia, lymphoma, or multiple myeloma; nephrotic syndrome; solid organ transplant; sickle cell disease; or other hemoglobinopathies.

†
https://www.cdc.gov/vaccines/acip/recs/grade/downloads/acip-evidence-recs-framework.pdf

§
https://www.cdc.gov/vaccines/acip/recs/grade/about-grade.html

¶ Immunocompromising conditions are defined as chronic renal failure, nephrotic syndrome, immunodeficiency, iatrogenic immunosuppression, generalized malignancy, HIV, Hodgkin disease, leukemia, lymphoma, multiple myeloma, solid organ transplant, congenital or acquired asplenia, sickle cell disease, or other hemoglobinopathies.

** The case definition used by CDC’s Active Bacterial Core surveillance is isolation of *S. pneumoniae* from a normally sterile site or pathogen-specific nucleic acid in a specimen obtained from a normally sterile body site using a validated molecular test. https://www.cdc.gov/abcs/methodology/case-def-ascertain.html

†† Alcoholism; chronic heart, liver, or lung disease; cigarette smoking; or diabetes mellitus.

§§ https://www.cdc.gov/vaccines/acip/recs/grade/pneumo-PCV20-risk-based.html; https://www.cdc.gov/vaccines/acip/recs/grade/pneumo-PCV15-PPSV23-risk-based.html; https://www.cdc.gov/vaccines/acip/recs/grade/pneumo-PCV20-age-based.html; https://www.cdc.gov/vaccines/acip/recs/grade/pneumo-PCV15-PPSV23-age-based.html

¶¶ https://www.cdc.gov/vaccines/acip/recs/grade/pneumo-PCV20-risk-based-etr.html; https://www.cdc.gov/vaccines/acip/recs/grade/pneumo-PCV20-age-based-etr.html; https://www.cdc.gov/vaccines/acip/recs/grade/pneumo-PCV15-PPSV23-risk-based-etr.html; https://www.cdc.gov/vaccines/acip/recs/grade/pneumo-PCV15-PPSV23-age-based-etr.html

*** Serotypes 22F and 33F, in addition to PCV13 serotypes.

††† Serotypes 8, 10A, 11A, 12F, 15B, 22F, and 33F, in addition to PCV13 serotypes.

§§§ Serotypes 2, 8, 9N, 10A, 11A, 12F, 15B, 17F, 20, 22F, and 33F, in addition to PCV13 serotypes.

¶¶¶ Lower bound of the two-sided 95% CI of the OPA GMT ratio (PCV15 / PCV13) to be >0.5.

**** For PCV15-unique serotypes 22F and 33F, defined as the lower bound of the two-sided 95% CI of the OPA GMT ratio (V114 / PCV13) to be >2.0 and the lower bound of the two-sided 95% CI of the differences (V114 − PCV13) between the percentages of participants with a fourfold rise to be >0.1. For serotype 3, defined as the lower bound of the two-sided 95% CI of the OPA GMT ratio (V114 / PCV13) to be >1.2 and the lower bound of the two-sided 95% CI of the differences (V114 − PCV13) between the percentages of participants with a fourfold rise to be >0.

†††† Range reflects the difference in results across studies.

§§§§ Subjects with a fourfold or larger rise in OPA GMT titer postvaccination compared with prevaccination.

¶¶¶¶ Defined as the lower bound of the two-sided 95% CI of the ratio (PCV20 / PCV13) of opsonophagocytic geometric mean titers being >0.5.

***** Lower cost and improved health outcomes compared with previous recommendations.

††††† For adults who have received PCV13 but have not completed their recommended pneumococcal vaccine series with PPSV23, one dose of PCV20 may be used if PPSV23 is not available.
